# First Case of Pleural Empyema Caused by *Staphylococcus simulans*: Review of the Literature

**DOI:** 10.1155/2018/7831284

**Published:** 2018-10-11

**Authors:** Amos Lal, Jamal Akhtar, Ashfaq Ullah, George M. Abraham

**Affiliations:** ^1^Department of Medicine, Saint Vincent Hospital, 123 Summer Street, Worcester, MA 01608, USA; ^2^Department of Medicine, Division of Pulmonology and Critical Care Medicine, Saint Vincent Hospital, 123 Summer Street, Worcester, MA 01608, USA; ^3^Department of Medicine, Chief of Medicine, Saint Vincent Hospital, 123 Summer Street, Worcester, MA 01608, USA; ^4^Professor of Medicine, University of Massachusetts Medical School, Worcester, MA, USA; ^5^Governor, MA Chapter, and Regent, American College of Physicians (ACP), Philadelphia, PA, USA; ^6^Chair-Elect, Board of Governors (ACP), Philadelphia, PA, USA; ^7^Chair, Infectious Disease Board, American Board of Internal Medicine (ABIM), Philadelphia, PA, USA; ^8^Vice Chair, Board of Registration in Medicine (BORIM), Commonwealth of Massachusetts, Wakefield, MA, USA

## Abstract

*Staphylococcus simulans* is a coagulase-negative organism, mainly an animal pathogen. Reports of human infection have been infrequent, mainly in patients with repeated animal contact. We report the first case of pleural empyema in an elderly woman. *S. simulans* tends to cause more severe infection because of a biofilm layer which helps in adherence and colonization of smooth surfaces, especially prosthetic devices, shunts, and catheters. The challenging problem even after CoNS isolation and identification is the assessment of their clinical relevance. Major factors that inhibit the penetration of antibiotics is the large-sized effusions/empyema, thickness of pleura, and the nature of antibiotic itself. Source control for septic patients remains the cornerstone of treatment along with optimal antimicrobial coverage. *Staphylococcus simulans*, a coagulase-negative staphylococcus, is emerging as an important cause of virulent infections with high mortality in humans. Given its propensity for multidrug resistance, including vancomycin, there is an imperative for early and accurate identification of the isolate. Despite aggressive treatment, the patient succumbed to her illness.

## 1. Introduction

*Staphylococcus simulans* is a coagulase-negative staphylococcus. It is mainly an animal pathogen and has been found to cause bovine mastitis. It can occasionally colonize the human skin. Human infections with *Staphylococcus simulans* have rarely been reported, mainly in patients who have repeated contact with animals. We report the first case of pleural empyema caused by *S. simulans*.

### 1.1. Case Report

**Figure 1:**
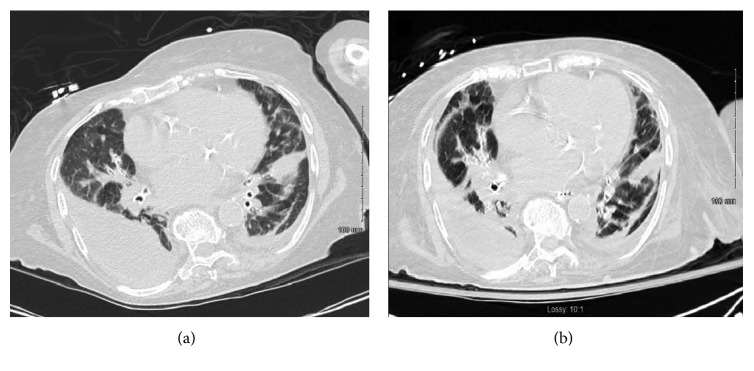
(a) CT chest showing bilateral empyema (right more than left). (b) CT chest showing persistent bilateral empyema after attempted drainage through chest drains.

An 80-year-old woman was admitted to the hospital after a fall. Her prior history was notable for coronary artery disease status post percutaneous intervention, poorly controlled type 2 diabetes mellitus, congestive heart failure, hypothyroidism, and atrial fibrillation. She had had multiple mechanical falls in the past with cervical spine and right-sided rib fractures. She had no recent hospitalization in the last 90 days and has been living at home prior to presentation. There was no history of exposure to the farm animals. During this hospitalization, she developed progressive dyspnea and hypoxia. Computed tomography (CT) revealed a bilateral pleural effusion, right more than left, with diffuse interlobular septal thickening. Note was also made of a diffuse, mosaic-like attenuation of the lung parenchyma, likely related to air trapping or obstructive small airway disease. There was no pleural enhancement, septations, or air noted within the pleural space **(**Figure 1(a)**)**. She was noted to have new fracture of right posterior seventh, eighth, and ninth ribs. Laboratory data at admission revealed a white blood cell (WBC) count of 9.7 × 1000/µL (with 83% neutrophils and 1% eosinophils). Her hemoglobin was 11.09 g/dL, hematocrit 40.6%, platelets 143 × 1000/µL, total protein 5.1 g/dL, blood urea nitrogen (BUN) 31 mg/dL, serum creatinine 1.51 mg/dL, serum sodium (Na) 145 mEq/L, chloride (Cl) 102 mEq/L, potassium (K) 3.6 mEq/L, aspartate aminotransferase (AST) 24 IU/L, and lactate dehydrogenase (LDH) 472 IU/L. Serum procalcitonin levels were elevated to 0.59 ng/mL (normal 0.00 – 0.08 ng/mL) and hemoglobin A1C (HbA1C) to 8.9%.

Blood cultures prior to initiation of antimicrobial therapy returned negative. Sputum culture was negative for bacteria, including acid-fast bacilli. Transthoracic echocardiography was unremarkable.

**Figure 2:**
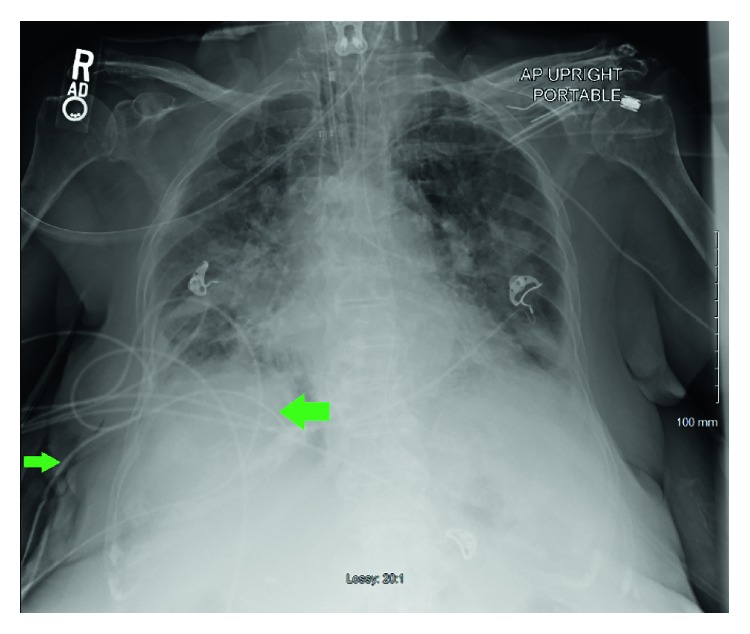
Chest X-ray showing the right-sided chest tube (green arrows), positioned in the most dependent area.

The patient was treated initially with levofloxacin and ampicillin/sulbactam for a pneumonia and parapneumonic pleural effusion. Despite antimicrobial therapy, her respiratory status continued to deteriorate and within 48 hours of hospitalization required endotracheal intubation and ventilatory support. A thoracentesis yielded 250 ml of pus (WBCs more than 50,000 with 95% neutrophils, elevated protein 3.6 g/dL (normal 0.0–2.4 g/dL), LDH 13461 IU/L, glucose 6 mg/dL, and pH 6.94). A right-sided thoracostomy was performed to facilitate evacuation of the empyema using a 32 French (F) thoracostomy tube. Postprocedure chest X-ray confirmed the optimal placement of the tube **(**Figure 2**)**. Gram stains obtained from the pleural fluid collected under sterile conditions revealed gram-positive cocci, and her antibiotics were changed to vancomycin. The final isolate on culture and sensitivity was identified as *S. simulans* with heavy growth, susceptible to vancomycin and clindamycin only. A repeat CT scan of the chest revealed a persistent collection of right-sided empyema **(**Figure 1(b)**)**. Ultrasound imaging of the pleural collections did not demonstrate any septations or loculated pockets on either side. Second attempt was made to evacuate the pleural collection with the use of intrapleural fibrinolytic tissue plasminogen activator (t-PA) with little success. The patient was considered a high-risk candidate for surgical intervention considering her frailty and other medical comorbidities. Her course was further complicated by circulatory shock requiring vasopressor supports and atrial fibrillation with rapid ventricular response requiring multiple rate-controlling drugs. After about 3 weeks of a tenuous course, her family elected to withdraw care and she passed away very shortly thereafter.

## 2. Discussion

Coagulase-negative staphylococcus (CoNS) has become an important cause of nosocomial infections. Common human isolates are *Staphylococcus epidermidis*, *Staphylococcus capitis*, *Staphylococcus hominis*, *Staphylococcus haemolyticus*, *Staphylococcus warneri*, *Staphylococcus caprae*, *Staphylococcus saccharolyticus*, *Staphylococcus pasteuri*, *Staphylococcus saprophyticus*, and *Staphylococcus lugdunensis* [1].

*S. simulans* commonly affects cows, sheep, goats, and horses. Human infections are rare, and the literature has commonly reported soft tissue infection, osteomyelitis, bacteremia, urinary tract infection, prosthetic joint infection, and native valve endocarditis [1–8] **(**Table 1**)**. A case of pneumonia was reported by de Jesus et al. which revealed *S. simulans* in blood culture and CoNS in sputum culture [2]. de Jesus et al. also reported a case of acute respiratory failure, ARDS with blood culture growing *S. simulans* [2]. To the best of our knowledge, this is the first case of pleural empyema caused by *S. simulans*. In our patient, there was no history of contact with animals. Mode of acquisition of infection in our patient remained unclear.

*S. simulans* tends to cause more severe infection because of a biofilm layer which helps in adherence and colonization of smooth surfaces, especially prosthetic devices, shunts, and catheters [8]. *S. simulans* has also been shown to share virulence factors with *S. aureus* in infectious animal isolates, including staphylococcal enterotoxins, tissue necrosis cytotoxin Panton–Valentine leukocidin, and the methicillin-resistance gene, mecA [5]. The encapsulated forms of *S. simulans* (i.e., slime layer or biofilm) have an antiphagocytic effect on human polymorphonuclear leucocytes as compared to unencapsulated forms of the pathogen [8]. Sequencing of the tuf gene has been shown to be the most accurate for the species identification of CoNS [9].

The critical event in the establishing the pathogenicity includes formation of multilayered biofilm, especially in foreign body-associated infections caused by CoNS. Members of the genus Staphylococcus produce various proteinaceous and nonproteinaceous adhesins, to mediate attachment to host surfaces, such as plasma extracellular and matrix proteins or even host cells [10, 11].

There are limited data available about how CoNS which are usually common pathogens in veterinary medicine are gaining pathogenicity in human hosts. There are some shared virulence factors with *Staphylococcus aureus* which are documented in Table 2 [5, 12–14]. Of note, mecA-positive CoNS are capable to horizontally transfer their genes inside the staphylococcal genus with the prospective to contribute increase in new methicillin-resistant strains [12].

The challenging problem even after CoNS isolation and identification is the assessment of their clinical relevance. The major diagnostic question remains as to whether the CoNS isolate represents a contamination of the specimen, physiological colonization of the skin or mucus membranes, or a clinically significant infection. In our case, the absence of other microbiological data and the confidence in the sterile process of procuring the sample from pleural fluid warranted consideration of *S. simulans* in the pleural fluid as pathogenic. Important considerations include isolation of a strain in pure culture from the site of infection and repeated isolation of the same strain over the course of infection [11, 15].

While treating the pleural infections, clinicians need to be cognizant of the choice of antimicrobial therapy and relevant pharmacokinetics. Major factors that inhibit the penetration of antibiotics is the large-sized effusions/empyema, thickness of pleura, and the nature of antibiotic itself; acute inflammation is proven to be a supporting factor due to vasodilation [16, 17]. Teixeira et al. demonstrated that penicillin has the best penetration for the treatment of pleural pathologies, followed by metronidazole; gentamicin was found to have poor penetration and considered a poor choice for treatment of empyemas [16].

*S. simulans* is known to demonstrate resistance to multiple antibiotics including vancomycin [2]. In our case, the organism was resistant to ampicillin/sulbactam, cefazolin, ciprofloxacin, oxacillin, and tetracycline, but it was susceptible to vancomycin in vitro. The use of vancomycin in pleural infections has long been debated due to its suboptimal penetration; however, in our case, the multidrug-resistant nature of the isolate required its use. It is debatable in retrospect if continuation of a broader antimicrobial coverage would have changed the outcome for the patient, considering poor biochemical and clinical response with the chosen antibiotic regimen.

Source control for septic patients remains the cornerstone of treatment along with optimal antimicrobial coverage. The MIST-2 study reiterated the fact where use of t-PA and DNase combination therapy in patients with pleural infection improved drainage of pleural empyema, resulting in reduction in hospital stay and need for thoracic surgery intervention [18].

## 3. Conclusion

*Staphylococcus simulans*, a coagulase-negative staphylococcus, is emerging as an important cause of virulent infections with high mortality in humans. Given its propensity for multidrug resistance, including vancomycin, there is an imperative for early and accurate identification of the isolate.

## Figures and Tables

**Table 1:** 

Author	Age/sex	Diagnosis	Tissue culture for *S. simulans*	Blood culture for *S. simulans*	Antibiotic resistance	Outcome	Animal exposure
Shields et al. [5]	80/M	Right great toe cellulitis	Positive	Not specified	Tetracycline resistance	Resolution with TMP-SMX	Not specified
Tous Romero et al. [6]	60/M	Pyoderma left hand	Positive	Not specified	Not specified	Resolution with azithromycin	Positive
Al Kline et al. [8]	65/M	Abscess, osteomyelitis right foot	Positive bone culture	Positive	Ampicillin, ciprofloxacin, clindamycin, oxacillin, penicillin, ceftriaxone	Resolution with IV vancomycin	Positive
Vallianou et al. [3]	46/M	Vertebral osteomyelitis, native valve, endocarditis	Not specified	Positive	Methicillin	Resolution with IV vancomycin, teicoplanin, oral clindamycin	Positive
Sturgess et al. [7]	77/F	Right pubic osteomyelitis	Not specified	Positive	Pan-sensitive	Resolution with flucloxacillin, fusidic acid	Not specified
de Jesus et al. [2]	84/M	Septicemia, colon cancer	Not specified	Positive	Methicillin-sensitive	Died	Not specified
de Jesus et al. [2]	41/M	Acute respiratory failure, ARDS, H/O HIV, IV drug abuse	Not specified	Positive	Methicillin-sensitive	Died	Not specified
de Jesus et al. [2]	63/M	Pneumonia	CN staph in sputum	Positive for *S. simulans*	Not specified	Resolved with erythromycin, cefuroxime	Not specified
de Jesus et al. [2]	58/M	Colon cancer, septicemia	Not specified	Positive	Methicillin-resistant	Resolved with vancomycin	Not specified
Males et al. [1]	39/M	Right ankle, osteomyelitis, septic, arthritis	Positive	Positive	Pan-sensitive	Penicillin	Not specified

**Table 2:** 

Staphylococcal species	Common virulence factors	Clinical manifestations
*S. aureus and S. simulans*	Staphylococcal enterotoxins (*se*)	Gastrointestinal manifestations of diarrhea, nausea, vomiting, and enterocolitis.
	Tissue necrosis cytotoxin Panton–Valentine leukocidin (*pvl*)	Hospital-acquired pneumonia, infective endocarditis, and tissue necrosis
	Methicillin-resistance gene (*mecA*)	Major contributing factor for increase in new methicillin-resistant strains
	Exfoliative toxins (*eta, etb*)	Cutaneous manifestations of cellulitis
	Toxic shock syndrome toxin-1 (*tst*)	Septic shock and disseminated blood stream infections
